# The Finding of the Severe Acute Respiratory Syndrome Coronavirus (SARS-CoV-2) in a Wild Eurasian River Otter (*Lutra lutra*) Highlights the Need for Viral Surveillance in Wild Mustelids

**DOI:** 10.3389/fvets.2022.826991

**Published:** 2022-03-31

**Authors:** Miguel Padilla-Blanco, Jordi Aguiló-Gisbert, Vicente Rubio, Víctor Lizana, Eva Chillida-Martínez, Jesús Cardells, Elisa Maiques, Consuelo Rubio-Guerri

**Affiliations:** ^1^Department of Pharmacy, Facultad de Ciencias de la Salud, Universidad Cardenal Herrera-CEU, Valencia, Spain; ^2^Servicio de Análisis, Investigación, Gestión de Animales Silvestres (SAIGAS), Facultad de Veterinaria, Universidad Cardenal Herrera-CEU, Valencia, Spain; ^3^Instituto de Biomedicina de Valencia del Consejo Superior de Investigaciones Científicas, Centro de Investigació Biomédica en la Red sobre Enfermedades Raras, Instituto de Salud Carlos III, Valencia, Spain; ^4^Wildlife Ecology & Health Group (WE&H), Universitat Autònoma de Barcelona (UAB), Barcelona, Spain; ^5^Department of Biomedical Sciences, Facultad de Ciencias de la Salud, Universidad Cardenal Herrera-CEU, Valencia, Spain

**Keywords:** SARS-CoV-2, RT-PCR, *Lutra lutra*, wildlife, Spain

## Abstract

Animals have been involved in the three known outbreaks of severe respiratory syndromes due to coronaviruses (years 2005, 2012, and 2019). The pandemic nature of the SARS-CoV-2 outbreak increases the likelihood of infection from humans of susceptible animal species that, thus, could become secondary viral hosts and even disease reservoirs. We present evidence of spillover infection of wild mustelids by reporting the presence of SARS-CoV-2 in a Eurasian river otter found near a water reservoir in the Valencian Community (Spain). We detected the virus using two different commercial RTqPCR assays on RNA extracted from the nasopharynx (swabbing) and from lung tissue and mediastinal lymph node homogenates. The corresponding samples from two additional otters from distant sites tested negative in identical assays. The diagnosis in the positive otter was confirmed by two-tube RT-PCR assay in which RNA was first retrotranscribed, and then specific regions of the spike (*S*), nucleocapsid (*N*), and *ORF10* genes were separately amplified from the produced cDNA, followed by electrophoretic visualization and Sanger sequencing. The sequences of the amplified products revealed some non-synonymous changes in the *N* and *ORF10* partial sequences, relative to the consensus sequence. These changes, identified already in human patient samples, point to human origin of the virus, although their specific combination was unique. These findings, together with our previous report of SARS-CoV-2 infection of feral American mink, highlight the need for SARS-CoV-2 surveillance of wild or feral mustelids to evaluate the risk that these animals could become SARS-CoV-2 reservoirs.

## Introduction

A zoonotic origin of the severe acute respiratory syndrome coronavirus 2 (SARS-CoV-2) (1) was suspected from the initial human cases (2), linked to the Huanan seafood market of Wuhan (Hubai province, China), where many types of animals were sold (1). SARS-CoV-2 was initially proposed (2, 3) to have evolved from RaTG13, a coronavirus sampled in 2013 in a bat (*Rhinolophus affinis*) from China with which the Wuhan strain of SARS-CoV-2 shared ~96.1% genome sequence identity (3). However, as the receptor-binding domain (RBD) of the spike protein (S) is crucial for infection [due to its interaction with the angiotensin-converting enzyme 2, ACE2, that is, the cellular receptor for SARS-CoV-2 (4)], the limited (85%) sequence identity (5) of the RBDs of SARS-CoV-2 and RaTG13 led to suspect the existence of an intermediate host. Previously, the severe acute respiratory syndrome coronavirus (SARS-CoV) and the Middle East respiratory syndrome coronavirus (MERS-CoV) had been shown to have emerged in bats but to have evolved in civets and camels, respectively, before causing human outbreaks in 2002 and 2013 (6, 7). For SARS-CoV-2, an initial proposal of the Malayan pangolin (*Manis javanica*) as an intermediate host (based on the ~97% sequence identity of the RBDs of the pangolin coronavirus with the Wuhan SARS-CoV-2) (8) lost ground because of low (91%) whole-genome sequence identity of these coronaviruses (5). A closer relative to the initial Wuhan strain of SARS-CoV-2 may be BANAL-52, a coronavirus recently found in cave bats from North Laos (near China), which exhibits a whole-genome sequence identity with Wuhan SARS-CoV-2 similar to that of RaTG13 and a much higher identity (almost complete identity) in RBD sequence (9).

Whichever the animal origin of SARS-CoV-2 is, animals are also important in the context of the SARS-CoV-2 pandemic because some species could become secondary hosts of the virus following transmission from humans. This human-to-animal transmission has been recorded for pets and captive or farmed animals such as dogs (*Canis lupus familiaris*) (10), felines, such as cats (*Felis silvestris catus*) (11), lions (*Panthera leo*), and tigers (*Panthera tigris*) from zoos (12), and several species of mustelids (13–18). In experimental infections (19), pigs and chickens appeared non-susceptible, fruit bats became transiently infected, and ferrets presented subclinical infection. The infection of wild animals is of concern, as it may escape monitoring, with the possibility of viral evolution and re-entry into the human population from wild animal reservoirs. This is illustrated in the evolution of SARS-CoV-2 during the pandemic toward mice infectiousness, leading to the hypothesis of a mice origin of the omicron variant (20). Thus, the One Health concept should also consider SARS-CoV-2 surveillance in wild animals. The present article supports the need for this surveillance. This need was already highlighted by our prior report (21) of SARS-CoV-2 infection in feral American mink (*Neovison vison*) and has been stressed by the recent report of SARS CoV-2 among free-ranging white-tailed deer near Denver (22). Mustelids are believed to be highly susceptible to SARS-CoV-2 infection (14–19, 23). Thus, surveillance on wild otter also appeared pertinent, particularly since there is a report of captive Asian small-clawed otter (*Aonyx cinereus*) infection by SARS-CoV-2 in a North-American aquarium (Georgia, USA) (24). With the goal of contributing to the knowledge of the extent of SARS-CoV-2 infection in wild or feral mustelids, we now report the presence of SARS CoV-2 in a wild Eurasian otter (*Lutra lutra*) in the Valencia province of Spain. We document the presence of SARS-CoV-2 in the airway, the lungs, and a mediastinal lymph node of this animal. Our findings extend to a second otter species the susceptibility to infection by SARS-CoV-2, and they support the need for viral monitoring in feral and wild mustelids including otters.

## Materials and Methods

### Sample Collection

**Figure 1:**
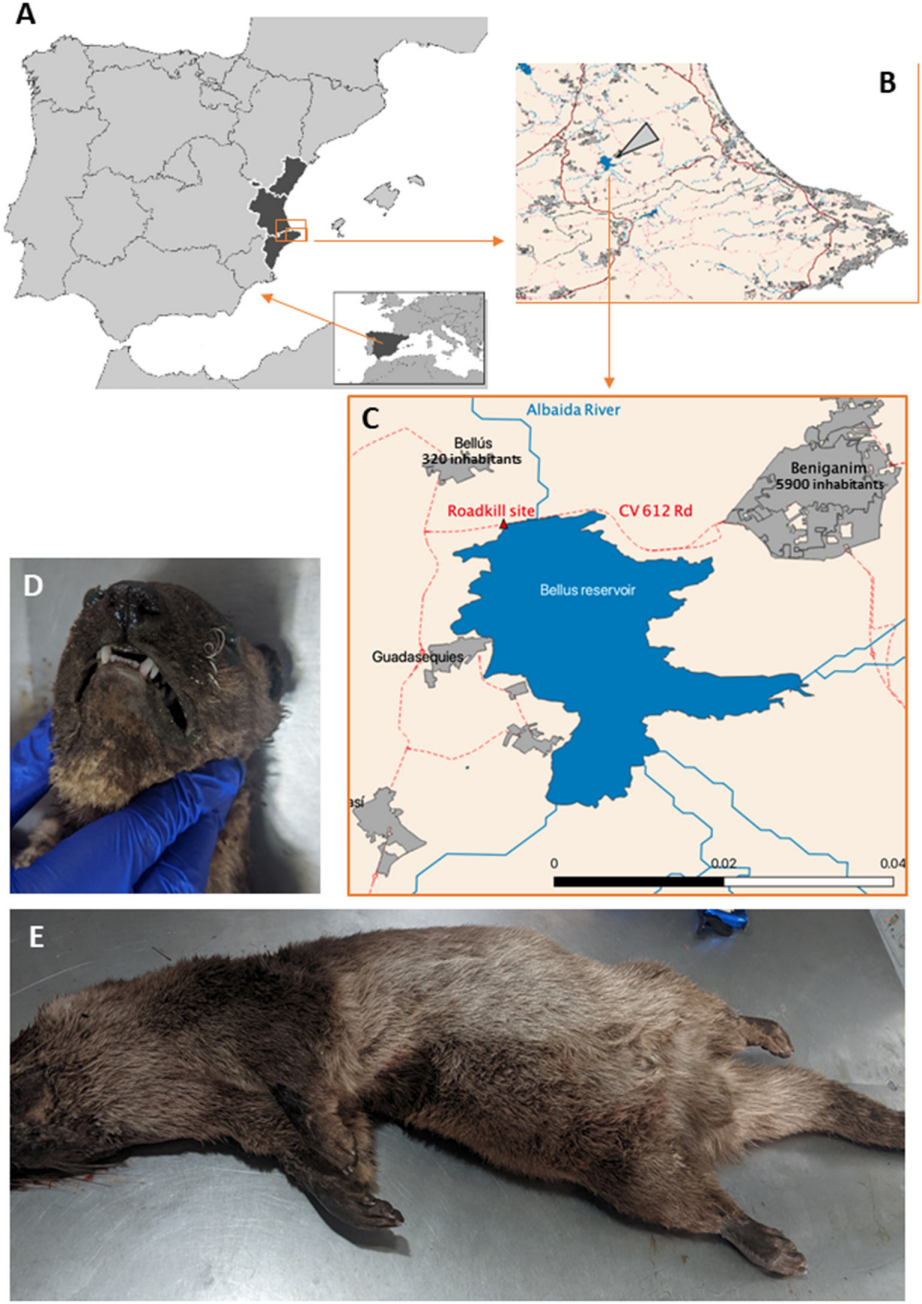
Otter carcass retrieval site and external aspect of the animal. **(A)** Europe (inset) and (main figure) Iberian Peninsula (Spain and Portugal) with the Valencian region in black, enclosing in a rectangle the area that is enlarged in **(B)**, where the large arrowhead points to the Bellús reservoir, **(C)** zooming on it and its surrounding area, marking the site where the animal was found. **(D,E)** Shows, respectively, views of the head and the remainder of the body of the animal.

The positive animal (from now on index case) was an adult male Eurasian river otter (*Lutra lutra*). The animal was found on August 25, 2021, freshly road killed (but not smashed) in a road near the Bellús reservoir of the Albaida river, in the part belonging to the village of Beniganim (population, 5,899 inhabitants; GPS coordinates, 30S 718216.80 4313010.69) [Figure 1; (25)]. Since 2015, otters have recolonized this reservoir (26), found in a relatively heavily populated territory (110 inhabitants per km^2^) in inland Valencia (a Mediterranean Spanish province) (25). The dead animal was preserved frozen (−20°C) until necropsy, performed 2 weeks later by a SARS-CoV-2-negative (RTqPCR of nasopharyngeal samples) staff professional from a wildlife surveillance center. The otter presented occipital fracture, splenic laceration with abdominal hemorrhage, and heterogeneous lung discoloration assumed to be also due to trauma. There were no noticeable macroscopic alterations in the respiratory organs. A nasopharyngeal swab, lung tissue, and a mediastinal lymph node were taken and placed aseptically in plastic tubes containing Sample Preservation Solution (reference number P042T0020100) from JiangSu Mole Bioscience (Taizhou, China; sold in Spain by Palex Medical, Madrid, Spain). This proprietary commercial solution contains trisodium citrate dyhydrate, guanidine thiocyanate, acetic acid, sodium acetate trihydrate and water buffer, and is used to inactivate the virus while preserving the viral RNA. The hermetically sealed tubes, placed at −80°C <2 h after procurement, remained at this temperature until their use in SARS-CoV-2 analyses.

Two additional river otters found at other locations of the Valencian Community were also tested. They were juvenile females. Their dates and sites of collections were July 7 at El Hondo, Elx (38°10′55″N 0°45′09″W) and August 2 at the Mijares River, Almassora (39°57′11.77″N, 0°4′16.18″W). Their collection sides were >100 km apart one from the other and from the site of collection of the positive otter (near Beniganim, 38°56′38″N, 0°26′38″W) in independent watercourses. They were kept frozen until nasopharyngeal, lung, and mediastinal samples were collected for the positive otter.

### RNA Extraction

Approximately 100 mg of lung tissue and the entire mediastinal lymph node were defrosted, and immediately homogenized in 0.2 ml of Sample Preservation Solution (see above), using hypochlorite-treated and autoclaved (121°C, 15 min) small-sized glass Dounce-type homogenizers. RNA in each of these entire homogenates as well as in 0.2 ml of Sample Preservation Solution, in which the nasopharyngeal swab had been placed, was isolated in 50 μl of RNase-free water using the NZY Total RNA Isolation kit (NZYtech, Lisboa, Portugal) and stored at −80°C. We repeated RNA extraction on a different day with the swab fluid and the lung tissue, to use the new lots of RNA in replicate analyses. The same procedures and instrumentation (including the use of the same homogenizers, duly cleaned, hypochlorite treated, and autoclaved after each utilization) were employed for processing tissue samples from the positive otter and from the two negative ones.

### Viral Detection and cDNA Preparation

We used for viral detection two one-tube RTqPCR commercial assays intended for human nasopharyngeal samples. One of them (Viasure, from CerTest Biotec, Zaragoza, Spain; sold by Palex Medical) (27) uses 5 μl of the isolated RNAs, amplifying regions of the *ORF1ab* and nucleocapsid (*N*) viral genes, as well as the host *RNaseP* gene. This last gene, used as an internal operational control gene, proved positive in otter as it previously was in American mink (21). Diagnosis was confirmed using the commercial NZYtech SARS-CoV-2 One-Step RT-PCR kit, *RdRp* and *N* genes, CE-IVD, utilizing 8 μl of the RNA preparations. This test uses the same fluorophore for measuring the combined signal from the *RdRp* and *N* viral genes, with control *RNaseP* detection with a second fluorophore.

For sequence information gathering, we amplified specific regions (Table 1) from the *S, N*, and *ORF10* viral genes, using appropriate primers in a two-tube RT-PCR procedure (21) applied to 8 μl of RNA isolated from the mediastinal lymph node (chosen because its viral level, assessed in conventional one-tube assays, was the highest; see Results Section). cDNA was first generated with NZY First-Strand cDNA Synthesis kit (NZYtech). Then we amplified separately the three selected gene regions in a mixture containing 10 μl of NZYSpeedy qPCR Green Master Mix (2 ×) (NZYtech), 400 nmol of each primer (Table 1), and 2 μl of the cDNA solution (final volume, 20 μl). The temperature protocol was (i) 2 min at 95°C; (ii) 40 cycles of a sequence of 5 s at 95°C and 30 s at 60°C; (iii) 30 s at 95°C; (iv) 30 s at 65°C; (v) 30 s at 95°C. Generation of the expected amplification product (Table 1), monitored by SYBR green fluorescence, was proven by electrophoretic visualization of DNA in agarose gels. We routinely use these primers (Table 1) in the same two-tube RT-PCR procedure applied to human viral samples, consistently yielding positive amplifications for 10^3^ viral genome copies/μl (inferred from Ct values in the Viasure test). They are highly specific, giving negative results with otter and human samples that tested negative in the Viasure assay.

**Table 1:** Fragments of viral genes that were PCR-amplified and sequenced here.

**Gene**	**Primers used for PCR amplification^**a**^**	**Amplified region** ^**b**^		
		**Start position**	**End position**	**Amplicon size (bp)**
*S*	Forward: 5′-GGACCTTGAAGGAAAACAGG-3′ Reverse: 5′-GAACCATTGGTAGATTTGCCA-3′	22,160	22,239	80
*N*	Forward: 5′-GCAGTCAAGCCTCTTCTCGT-3′ Reverse: 5′-TTGAACCAGCTTGAGAGCAA-3′	28,871	28,964	94
*ORF10*	Forward: 5′-ATTGCAACAATCCATGAGCA-3′ Reverse: 5′-TAGGGAGGACTTGAAAGAGCC-3′	29,556	29,704	149

*^a^Primers were designed with Primer3 (https://primer3.ut.ee/).*

*^b^Start and end positions are consensus genome coordinates for severe acute respiratory syndrome coronavirus 2 (SARS-CoV-2) [GenBank (NC_045512.2)]*.

In all the RTqPCR assays (commercial or two tube), we carried out in parallel cross-contamination controls and positive controls (B.1.177 variant of SARS-CoV-2), yielding in all cases respective negative and positive results. We used for all PCR procedures an Aria Mx Real-Time PCR (qPCR) instrument (Agilent Technologies, Santa Clara, CA, USA). The researchers performing all the steps from extraction to detection were SARS-CoV-2 negative.

### Sequencing and Data Analysis

Automated Sanger sequencing was done by a dedicated service (Genomics Department, Príncipe Felipe Research Centre, Valencia, Spain), using an ABI Prism 3730 sequencer (Applied Biosystems, Foster City, CA, USA), utilizing as sequencing primers the oligonucleotides used to amplify gene fragments.

*S, N*, and *ORF10* gene sequences were deposited in GenBank/EMBL/DDBJ (respective accession numbers OK235485, OK235486, and OK235487) and used for identification of related SARS-CoV-2 sequences in GenBank and GISAID databanks by searching with BLASTN (http://blast.ncbi.nlm.nih.gov). The BioEdit ver. 7.2.5 software (28) was used for nucleotide and corresponding amino acid sequence alignment and identity estimation, always including in the alignments the consensus early Wuhan sequence (GenBank NC_045512.2) as reference for identification of non-synonymous substitutions. For phylogenetic analyses, distance matrices were calculated, and tree topology was inferred by the maximum likelihood method based on p-distances (bootstrap on 2,000 replicates, generated with a random seed) using the MEGA X software (29).

## Results

We first tested RNA from the nasopharynx (swab) of the index case with the Viasure assay and consistently and repeatedly obtained positive results. The tests were positive on two independent RNA extractions from the swab fluid. We then also used lung tissue with positive results with the same test, repeating the lung tissue RNA extraction and again getting positive results. The mediastinal lymph node was the less abundant material, allowing a single RNA extraction (see the Materials and Methods Section), and was tested last, yielding, as the other samples, positive results in two rounds of Viasure testing, actually at an earlier cycle in the real-time PCR assay than with the swab and lung samples. All negative controls carried out simultaneously yielded negative results. Similar samples from two additional otters from other locations of the Valencian Community (see Sample Collection in the Materials and Methods Section) gave negative results, while samples from our positive animal run in parallel tested positive.

Results were also positive for all the RNA samples from the index case when we used another commercial RTqPCR test (from NZYtech) that does not completely overlap with the Viasure test in the genes detected (see the Materials and Methods Section), while the results were negative with the RNAs from the two additional otters tested in the same way.

In summary, in the positive animal, the RNAs extracted from the three samples were positive for the *ORF1ab* and *N* viral genes and the internal control (*RNaseP*) monitored in the Viasure test, with mean Ct values for *ORF10/N* viral genes of 36.0/31.7, 34.0/30.9, and 31.3/29.6 for lung tissue, nasopharyngeal swab, and lymph node RNA, respectively. The results of the NZYtech assay confirmed the same scale of relative abundance of SARS-CoV-2 RNA, with Ct values of 33.1, 30.9, and 29.3 for, respectively, lung tissue, nasopharyngeal swab, and lymph node RNAs. In the case of the other two otters, there was no amplification in these tests for as long as 40 cycles.

We obtained some sequence information on three regions of the *S, N*, and *ORF10* genes of the virus, utilizing the RNA extracted from the lymph node of the positive animal in a two-tube RT-PCR procedure [(21); see the Materials and Methods Section]. Gel electrophoresis revealed DNA products of the expected sizes, while Sanger sequencing confirmed that the amplified DNAs corresponded to the intended gene regions. Relative to the consensus Wuhan sequence (GenBank NC_045512.2), there were none, and six and one nucleotide changes in the partial *S, N*, and *ORF10* gene sequences, respectively. These substitutions (Table 2) caused four non-synonymous codon changes, three in *N* and one in *ORF10*. Of the viral genome sequences deposited in GISAID (https://www.gisaid.org/, accessed on October 18, 2021), 795 (195 of them from Europe) combined the partial *S* and *N* sequences found in the otter virus. The partial *ORF10* sequence found in the otter is found in thousands of deposited viral genomes (most of them related to the B.1.177 variant that was widely distributed in Europe). However, no GISAID-deposited viral genome sequence that combined the three partial sequences of *S, N*, and *ORF10* was found in the viral RNA obtained from this positive otter, indicating viral evolution not identified earlier.

**Table 2:** Differences in the partial gene sequences found here with the SARS-CoV-2 reference sequence.

**Viral gene**	***S***	***N***			***ORF10***
**Fragment (nucleotides)^**a**^**	**22,160–22,239**	**28,871–28,964**			**29,956–29,704**
**GenBank identifier**	**OK235485**	**OK235486**			**OK235487**
**Nucleotide changes** ^**a**^	None	28,877–28,878 AG>TC	28,881–28,883 GGG>AAC	28,899 G>T	29,645 G>T
**Amino acid changes** ^**b**^	None	Arg203Lys	Gly204Arg	Arg209Ile	Val30Leu

*^a^All the nucleotide positions refer to the whole-genome reference sequence NC_045512.2.*

*^b^Amino acid positions are those for the encoded protein*.

**Figure 2:**
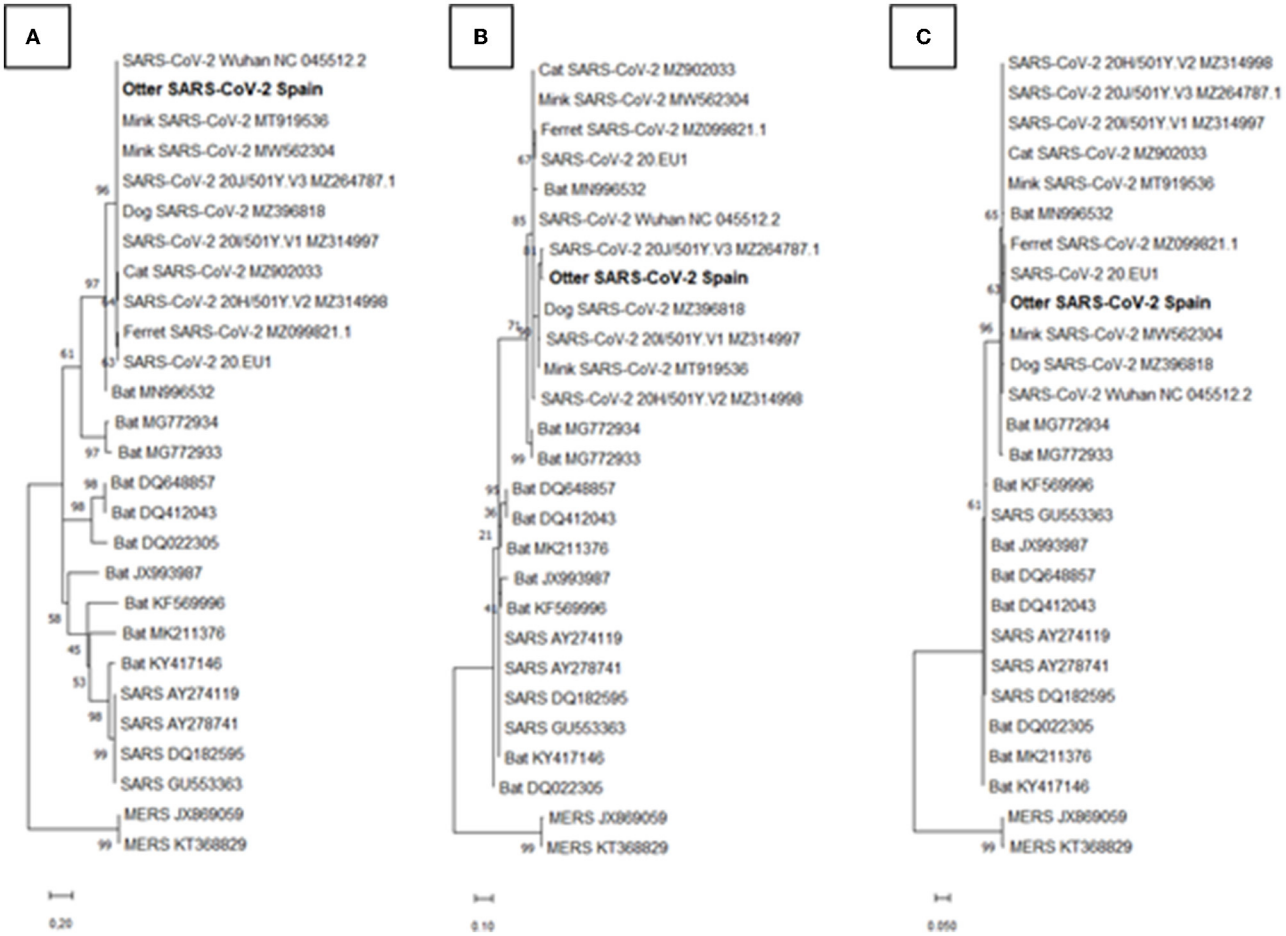
Molecular phylogenetic analyses based on partial gene sequences. These analyses used our sequences of 80-, 94-, and 149-nucleotide fragments of the severe acute respiratory syndrome coronavirus 2 (SARS-CoV-2) *S* gene **(A)**, *N* gene **(B)**, and *ORF10* gene **(C)** (respective consensus genome coordinates; GenBank acc number NC_045512.2, nt 22,160–22,239, 28,871–28,964, and 29,556–29,704). The trees include the corresponding sequences in SARS-CoV (SARS), Middle East respiratory syndrome coronavirus (MERS-CoV) (MERS), some bat coronaviral sequences, and SARS-CoV-2 sequences reported in animals or in the original Wuhan isolate and a few subsequent variants. The evolutionary history was inferred using the maximum likelihood method based on the Tamura–Nei model. In each case, the tree with the highest log likelihood (−722, −377.01, and −446.87, for trees of *S, N*, and *ORF10* genes, respectively) is shown. Initial tree(s) for the heuristic search were obtained automatically by applying the neighbor-join and BioNJ algorithms to a matrix of pairwise distance estimated using the maximum composite likelihood (MCL) approach and selecting the topology with superior log likelihood value. The trees are drawn to scale, with branch lengths according to the number of substitutions per site (see scale bar at the bottom). The sequences obtained from the Eurasian otter reported here is highlighted in bold-type. GenBank accession numbers for the different sequences are given at the end of each branch name. GenBank accession numbers for our otter viral sequences are OK235485, OK235486, and OK235487 for the *S, N*, and *ORF10* genes, respectively.

The three partial *S, N*, and *ORF10* gene sequences clustered with SARS-CoV-2 sequences (p-distances of around 0) in phylogenetic trees built as indicated in the Materials and Methods Section (bootstrap on 2,000 replicates from a random seed) (Figure 2). Other coronaviruses, such as SARS-CoV and MERS-CoV, formed different clusters. Although the small size of the sequenced regions limits the value of detailed quantitative comparisons based on p-distance values, p-distances of the partial gene sequences, relative to those of SARS-CoV and MERS-CoV, were much larger for *S* (13.1 and 18.2, respectively) than for *N* (0.2 and 0.45) and *ORF10* (0.07 and 0.67).

## Discussion

To date, there is only one report of SARS-CoV-2 infection of otter, affecting captive Asian small-clawed otters (*Aonyx cinereus*) at an aquarium in Georgia (USA). We now report the finding of SARS-CoV-2 in a wild otter belonging to a different genus and species, the Eurasian river otter (*Lutra lutra*), suggesting that all otter species may be susceptible to SARS-CoV-2. Our positive otter did not present obvious signs of disease, in line with our finding of relatively low viral loads in the samples from the nasopharynx, lungs, and mediastinal lymph node. The susceptibility to infection of otter by SARS-CoV-2 is not surprising, as otters belong to the mustelid family, and other mustelids, particularly minks and ferrets, have been proven to be quite susceptible to SARS-CoV-2 infection (14–19, 21), having forced entire mass culling of whole mink farms that were suffering from the infection. Although in most cases infected human caretakers caused the initial animal contagion, these animals effectively transmit the infection among themselves and even back to humans (15–17). All these characteristics make of mustelids potential secondary hosts of SARS-CoV-2, with the possibility of back passage of the virus between species and humans.

Until now, worries about these animals in the context of the SARS-CoV-2 pandemic have centered on pet and farmed mustelids, such as ferrets and mink (13–18). However, we already found two SARS-CoV-2-infected feral mink that were living in the wild (21). We now extend our observation to a Eurasian river otter living far away from the locations where these minks were found, suggesting multiple infecting events most likely derived from the universal expansion of the disease among humans. The fact that the time of the finding of the present infected otter took place when human 14-day notification rate of COVID-19 per 100,000 population in the region was above 1,000 cases (the fifth wave of COVID-19 in Spain)^1^ makes particularly plausible that the present positive otter could have been infected from humans. We previously reached the same conclusion in our report of infected feral mink, which occurred at an earlier peak of infections in the local human population (21). We ignore the transmission path from infected humans to these animals. In the case of the feral mink, we speculated on the possibility of water transmission. This possibility could fit otters, which also are aquatic animals, particularly since the Bellús reservoir is of low microbiological quality (30), with recorded episodes of urban wastewater discharge into it via the Albaida river (31) that have been reprimanded by the European Commission (32). In any case, the finding in our positive animal of viral RNA not only in the respiratory tract but also in the lung tissue, and in the highly secluded internally, mesenteric lymph node, excludes mere contact with external contaminated water that could carry viral remains as a reason for the positivity of this animal.

The finding that the exact combination of viral sequences identified here appears non-reported in any complete viral genome deposited in the GISAID database suggests viral evolution in infected individuals, either humans or animal hosts, that had escaped detection. Unnoticed evolution in humans is possible, since Spain does not stand out because of its frequency of whole SARS-CoV-2 genome sequencing, particularly during large disease peaks (33). Our findings call for continued surveillance of wild or feral susceptible animals, in particular, of mustelids. Our observations of two minks infected among a sample of 13 feral minks (21), and now of one infected individual among a sample of three wild otters warrant further study among the feral and wild populations of these animals.

## Data Availability Statement

The datasets presented in this study can be found in online repositories. The names of the repository/repositories and accession number(s) can be found in the article/supplementary material.

## Ethics Statement

The animal study was reviewed and approved by Animal Ethics Committees of UCH-CEU University (research permit no. CEEA 21/007).

## Author Contributions

CR-G and EM conceived the study. JA-G, VL, EC-M, and JC procured the samples. MP-B performed the molecular analyses. CR-G, EM, and MP-B analyzed the results. EM, VR, and CR-G were responsible for writing the article, although all authors contributed to this task, making substantial intellectual contributions, having read, corrected, and approved the manuscript.

## Funding

This research received external funding to CR-G and EM from Agencia Valenciana de Innovación: COVID-19. Ayudas de concesión directa a soluciones científico-innovadoras directamente relacionadas con la lucha contra la COVID-19, (Ref COVID-19-203) and from the Conselleria de Innovación Universidades, Ciencia y Sociedad Digital: Subvenciones a grupos de investigación emergentes (Ref, GV/2021/163), and from a grant to VR (PID2020-120322RB-C21) from the Spanish Agencia Estatal de Investigación (Plan Estatal de I+D+i).

## Conflict of Interest

The authors declare that the research was conducted in the absence of any commercial or financial relationships that could be construed as a potential conflict of interest.

## Publisher's Note

All claims expressed in this article are solely those of the authors and do not necessarily represent those of their affiliated organizations, or those of the publisher, the editors and the reviewers. Any product that may be evaluated in this article, or claim that may be made by its manufacturer, is not guaranteed or endorsed by the publisher.

## Footnotes

^1^ https://experience.arcgis.com/experience/42474fe756ee4714b7d8fbb730134b92
